# Moderators of the cost-effectiveness of transdiagnostic CBT for anxiety disorders over an 8-month time horizon using a net-benefit regression framework

**DOI:** 10.1186/s12913-023-09468-7

**Published:** 2023-06-08

**Authors:** Alexandra Chapdelaine, Helen-Maria Vasiliadis, Martin D. Provencher, Peter J. Norton, Pasquale Roberge

**Affiliations:** 1 PRIMUS Research Group, Faculty of Medicine and Health Sciences, Université de Sherbrooke, 3001 12e Avenue N, Sherbrooke, Québec J1H 5N4 Canada; 2 Department of Community Health Sciences, Faculty of Medicine and Health Sciences, Université de Sherbrooke - Campus de Longueuil, 150 Place Charles-Le Moyne, Longueuil, Québec J4K 0A8 Canada; 3 Centre de Recherche Charles-Le Moyne, 150 Place Charles-Le Moyne, Longueuil, Québec J4K 0A8 Canada; 4 École de psychologie, Université Laval, Pavillon Félix-Antoine-Savard, 2325 All. des Bibliothèques, Québec, Québec G1V 0A6 Canada; 5 The Cairnmillar Institute, 391-393 Tooronga Road, Hawthorn East, Victoria, VIC 3123 Australia; 6 Department of Family Medicine and Emergency Medicine, Faculty of Medicine and Health Sciences, Université de Sherbrooke, 3001 12e Avenue N, Sherbrooke, Québec J1H 5N4 Canada; 7 Centre de Recherche du Centre Hospitalier Universitaire de Sherbrooke (CRCHUS), 3001 12e Avenue N, Sherbrooke, Québec J1H 5N4 Canada

**Keywords:** Transdiagnostic, Cognitive behaviour therapy, Anxiety disorders, Group psychotherapy, Health care economics, Net-benefit regression framework

## Abstract

**Background:**

Access to evidence-based psychological treatment is a concern in many parts of the globe due to government-level financial constraints and patient-level barriers. Transdiagnostic cognitive behavioural therapy (tCBT) is an effective treatment approach that uses a single protocol for anxiety disorders which could enhance the dissemination of evidence-based psychotherapy. In a context of limited resources, the study of treatment moderators can allow to identify subgroups for which the cost-effectiveness of an intervention differs, information that could impact decision-making. So far, there has been no economic evaluation of tCBT for different subpopulations. The objectives of this study, using the net-benefit regression framework, were to explore clinical and sociodemographic factors as potential moderators of the cost-effectiveness of tCBT compared to treatment-as-usual (TAU).

**Methods:**

This is a secondary data analysis of a pragmatic randomized controlled trial opposing tCBT added to TAU (n = 117) to TAU only (n = 114). Data on costs from the health system and the limited societal perspectives, as well as anxiety-free days, an effectiveness measure based on the Beck Anxiety Inventory, were collected over an 8-month time horizon and used to derive individual net-benefits. The net-benefit regression framework was used to assess moderators of the cost-effectiveness of tCBT + TAU as opposed to TAU alone. Variables of sociodemographic and clinical nature were assessed.

**Results:**

Results showed that the number of comorbid anxiety disorders significantly moderated the cost-effectiveness of tCBT + TAU compared to TAU from the limited societal perspective.

**Conclusions:**

The number of comorbid anxiety disorders was identified as a moderator affecting the cost-effectiveness of tCBT + TAU compared to TAU from the limited societal perspective. More research is needed to strengthen the case of tCBT from an economic standpoint for large-scale dissemination.

**Trial registration:**

ClinicalTrials.gov: NCT02811458, 23/06/2016

**Supplementary Information:**

The online version contains supplementary material available at 10.1186/s12913-023-09468-7.

## Introduction

In reimbursement decisions and resource allocation, decision-makers often rely on data from economic evaluations, which is the comparative analysis of alternative interventions in terms of both their costs and consequences [1]. The incremental cost-effectiveness ratio (ICER) is a standard calculation in economic evaluations which represents the difference in mean cost (ΔC) between two interventions divided by the difference in mean effectiveness (ΔE). The ICER has several limitations, which can be addressed with the net-benefit regression framework [2, 3]. Notably, the information given by the ICER without a cost-effectiveness plane is limited in regard to its interpretability. A positive ICER can indicate a scenario where an intervention is less effective and less costly or a situation where an intervention is both more effective and costlier. From a statistical point of view, this also leads to interpretation issues when the uncertainty surrounding the ICER covers more than one quadrant on a cost-effectiveness plane or when comparing an ICER to a threshold [2, 3]. Further to this, researchers explain how the willingness to pay (WTP) is usually unknown [4, 5]. As has been described, the incremental net-benefit (INB) is a linear reformulation of the ICER that incorporates the WTP and is calculated as: λ*ΔE-ΔC, where λ is a willingness to pay threshold, and can be interpreted as cost-effective if positive [6, 7]. Exploring different variations of the WTP using the net-benefit approach allows for a better understanding of the effect of different WTP thresholds on the cost-effectiveness of an intervention [4, 5]. Moreover, the linear rearrangement of the ICER into the net-benefit allows for linear regression modeling and therefore offers the possibility to control for potential confounding factors and identify subgroups for which an intervention could be more or less cost-effective [3]. This approach can help inform decision-making in implementing an intervention or optimizing care trajectories when resources are limited, such as in the mental health sector.

Cognitive behavioural therapy (CBT) is the most recommended psychotherapy for anxiety disorders [8, 9]. Transdiagnostic cognitive behavioural therapy (tCBT) for anxiety disorders uses a single protocol to treat various anxiety disorders. It builds on the commonalities between recommended CBT protocols for anxiety disorders [10]. Some authors have highlighted the benefits of tCBT for anxiety disorders. In particular, they emphasize the reduced need for training for therapists who want to work with individuals with different type of anxiety disorders. It also aids the dissemination of group psychotherapy as this protocol allows for a heterogeneous clientele [10]. tCBT for anxiety disorders has been found effective in multiple meta-analyses when compared with passive as well as active controls such as diagnosis-specific CBT [11–15]. Determining the potential moderators of the cost-effectiveness of tCBT is important. Identifying moderators may provide information on subpopulations for which tCBT may be more or less cost-effective, optimizing health care trajectories.

The rare economic evaluations of psychotherapy for adults with common mental disorders that have used the net-benefit regression framework have adjusted their analytic models for factors including: total societal costs, age, sex, health-related quality of life, symptom severity, cancer, and comorbid neurological disorders at baseline [16, 17]. Evaluating the cost-effectiveness of CBT for anxiety disorders, Egger et al., (2015, 2016) adjusted their net-benefit regression model for gender, comorbid mental disorders, employment status, study sites, age, clinical index, baseline costs, and group psychotherapists delivering the intervention [18, 19]. None of these cost-effectiveness studies justified adjusting for these variables nor included an analysis of their potential impact as moderators.

While potential moderators relating to the cost-effectiveness of CBT are not well specified, predictors and moderators of treatment outcomes related to CBT for common mental disorders have included sociodemographic and clinical factors [20–23]. The literature however on the sociodemographic and clinical factors associated with tCBT-related outcomes is scarce. Recently, Gonzáles-Blanch et al., (2021) assessed general predictors and moderators of treatment outcome in tCBT compared to treatment-as-usual (TAU) for emotional disorders. In their exploratory analysis, among the sociodemographic factors, marital status and employment status moderated the association between the intervention and treatment outcome (score on the seven-item Generalized Anxiety Disorder scale (GAD-7)) [24]. The presence of anxiety and depression comorbidity and baseline severity moderated the association among the clinical variables assessed. Compared to TAU, tCBT had a greater positive effect in individuals with comorbidity and higher symptom severity at baseline. The intervention however seemed less beneficial for those taking antidepressants at baseline than those who did not [24]. As for related costs, some found a decrease in overall and mental health care costs after CBT for anxiety disorders, but potential moderators were not identified [25, 26].

A number of the studies on CBT and tCBT [18, 19, 24] took place in Germany and Spain, where although psychological services are covered or reimbursed by the healthcare system, long waiting lists are an issue. Similarly, in Quebec, Canada, where the public healthcare system covers most medical services for residents; long waiting lists have been described for psychological services [27]. For those who cannot afford private psychological services and rely on public services, timely access to care is of concern. In a context of limited financial and human resources [28], tCBT could prove beneficial [10]. In a recent paper, we showed that there was a probability of ≥95% that tCBT added to treatment as usual was cost-effective compared to treatment as usual from the health system and limited societal perspectives at a WTP of $25/anxiety-free day and $40/anxiety-free day over an 8-month time horizon, respectively [29]. Identifying subgroups however for which tCBT is cost-effective may further help optimize the allocation of resources and improve the value of mental health care. In this secondary data analysis, we therefore aimed to conduct a net-benefit regression analysis [3] to identify associated moderators of the cost-effectiveness of group tCBT [30] added to TAU compared to TAU only for adults with anxiety disorders in the province of Quebec, Canada.

## Methods

### Sample

The data used in this study are from a 12-month multi-centered, single-blinded, pragmatic randomized controlled trial, which aimed to evaluate the effectiveness of tCBT + TAU (n = 117) compared to TAU alone (n = 114) in the province of Quebec [31, 32]. Randomization was stratified 1:1 by study sites with blocking. The study took place in community-based care settings in three health administrative regions. Participants self-referred to the study through ads in social media, regional newspapers, and bulletin boards. The inclusion criteria were: (1) aged 18–65; (2) fluent in spoken and written French; and (3) meeting Diagnostic and Statistical Manual of Mental Disorders (DSM-5) [33] criteria for panic disorder (PD), agoraphobia (AGO), social anxiety disorder (SAD) and/or generalized anxiety disorder (GAD), with a clinical severity rating (CSR) ≥ 4 on the Anxiety and Related Disorders Interview Schedule for DSM-5 (ADIS-5) [34]. The principal anxiety disorder was defined as the one with the higher CSR rating. If there was a similar rating, clinical judgement was used to make the final decision. Exclusion criteria included the presence of either active suicidal intent, psychosis, bipolar disorder, substance-related and addictive disorders, cognitive impairment, and consultation with a psychiatrist in the past 12 months.

### Interventions

The tCBT intervention was provided during 12 weekly 2-hour group sessions co-led by two certified therapists. Groups generally included ten participants. The treatment protocol focused on CBT components: (1) education and self-monitoring, (2) specific cognitive restructuring, (3) graduated exposure and response prevention, and (4) generalized cognitive restructuring (i.e., focus on more general anxious style) [30]. Compared to conventional CBT, tCBT uses only one treatment protocol to simultaneously address a range of anxiety disorders by targeting common cognitive and behavioural processes with recurring elements of more specific CBT protocols [10].

Participants, both in the tCBT + TAU and TAU groups, given the pragmatic design, could stop or start any new treatment during the study period, and there was no restriction on the type of treatments received (e.g., psychotherapy [including CBT], pharmacotherapy, alternative and complementary medicines). For TAU, it was possible for participants to receive no treatment for their anxiety disorder.

### Economic evaluation

#### Data collection

For this analysis, three data points were considered over an 8-month time horizon: baseline (T_0_), post-treatment (T_1_: 4 months after randomization), and 4-month post-treatment (T_2_: 8 months after randomization). The time horizon being less than one year, no discount rates were applied. Data were collected during in-person (T_0,_ T_1_) and telephone (T_2_) assessments. All participants provided written consent.

#### Mental health service use and costs

The methodology for this economic evaluation has been described in detail elsewhere [29]. Briefly, health service use data for mental health reasons were collected retrospectively, over the past four months since the current assessment, with a structured interview guide [35]. Self-reported data on mental health related medical and social services used in an outpatient setting (in the public and the private sector), emergency department visits, inpatient stays, and outpatient medications delivered were considered as well as information on time spent on medical appointments, transportation to and from medical appointments, day-to-day assistance, work productivity (long-term sick leave and presenteeism) and the use of alternative and complementary medicine, all for mental health reasons. The cost of the tCBT intervention was estimated based on sessions being led by one therapist from the public sector and one therapist from the private sector. It included therapist fees (time in session and preparation, phone follow-up, individual pre-therapy appointment), general overhead costs, and supporting documents for participants.

The health system and the limited societal [36] perspectives were considered. No data was available on costs from the criminal justice and education sectors. For costs incurred by the public healthcare system, a direct allocation method was used based on annual financial and activity reports of health establishments in Quebec, submitted to the Quebec Ministry of Health and Social Services [37]. Costs were valued in 2020 Canadian dollars currency ($CAN) after adjustment with the Canadian consumer price index for January 2020 in Quebec [38]. For more detail on costing and cost data sources, see the Supplementary Methods Appendix 1.

#### Effectiveness outcome

Effectiveness was evaluated using Anxiety-Free Days (AFDs), a concept first introduced as an outcome for depression by Lave et al., (1998) [39]. It is a summary outcome of symptoms variation over time [39] that is valid and easily interpreted [40]. The Beck Anxiety Inventory (BAI) [41] was used to elicit AFDs as it is not specific to a particular anxiety disorder. An AFD value was computed at each assessment. Based on the BAI, a score of 7 or less, corresponding to the minimal anxiety cut-off, was considered a day free of anxiety (1 AFD). A score of 26 or more on the BAI (severe anxiety cut-off) was considered a day with anxiety (0 AFD). For scores in-between, values were weighted accordingly. Linear interpolation was used to assess the number of AFDs between each assessment (T_0_-T_1_, T_1_-T_2_). The number of AFDs per period was summed to cover the 8-month time horizon (T_0_-T_2_). Consequently, the range of possible AFDs was 0 to 244.

### Individual-level characteristics

Factors potentially associated with mental health-related costs (e.g., direct medical costs, indirect medical costs, and indirect costs including work productivity) and/or effectiveness of tCBT and CBT according to the literature were considered for this analysis. Sociodemographic factors considered included: age (continuous variable), sex (male, female), marital status (in a relationship, single/separated/widowed) [24], occupation (works full-time, works part-time, non-remunerated, sick leave) [24], education (high school or less, collegial or vocational, university) [42], and self-perceived economic status (at ease, sufficient, poor or worse) [42–48]. Health system factors considered included: complementary private health insurance (yes, no), covered under a private medication insurance plan (yes, no) [46], and having a family practitioner (yes, no) [49]. Clinical factors studied included: the presence of comorbid major depression (yes, no) [24, 50–52], the presence of comorbid anxiety disorders (none, one, two, three based on the inclusion criteria [GAD, SAD, AGO, PD]) [50, 51], anxiety symptom severity at baseline (continuous variable) [20, 21, 23, 24, 53], self-perceived mental health (excellent/very good, good, average or less) [54], self-perceived physical health (excellent/very good, good, average or less) [55, 56], taking psychotropic medication (yes, no) [24, 57] and other ADIS-5 mental health comorbidities (those with at least one count in the current sample: dysthymia, specific phobia, post-traumatic stress disorder, obsessive-compulsive disorder, major depression) (yes, no) [18, 19] were considered.

### Statistics

#### Missing data

On the assumption of a ‘Missing at Random’ mechanism, cost and clinical outcomes were imputed with Amelia II multiple imputation R package, which uses a bootstrapping-based expectation-maximization algorithm that is appropriate for longitudinal, continuous, and categorical data [58]. Variables with a skewed distribution were not transformed and the imputation model was not restricted by logical bounds [59–61]. Twenty datasets were imputed, and the imputation model included baseline variables associated with the probability of missing data. The pooling method for the linear regression estimates was based on Rubin’s rule and provided by SPSS [62].

#### Net-benefit regression framework

The net-benefit regression framework incorporates the notion of willingness to pay threshold (WTP, λ) in a linear reorganization of the ICER where the INB is equal to $$\widehat{{\mu }}$$ΔEλ-$$\widehat{{\mu }}$$ΔC. If the INB is positive, the new intervention is deemed cost-effective because the monetary value of its effect outweighs its cost at a determined WTP. Its statistical properties [2, 3] makes it suitable for linear regression analysis by defining an individual (_i_) net-benefit ($${\text{n}\text{b}}_{\text{i}})$$ for each participant: $$\widehat{{\text{n}\text{b}}_{\text{i}}}={{\lambda }\widehat{\text{E}}}_{\text{i}}-\widehat{{\text{C}}_{\text{i}}}$$. A basic linear net-benefit regression model would be: $$\widehat{{\text{n}\text{b}}_{\text{i}}}=\widehat{{\beta }}\text{I}\text{n}\text{t}\text{e}\text{r}\text{v}\text{e}\text{n}\text{t}\text{i}\text{o}\text{n}+{\alpha }$$, where $$\widehat{{\beta }}\text{I}\text{n}\text{t}\text{e}\text{r}\text{v}\text{e}\text{n}\text{t}\text{i}\text{o}\text{n}$$ is the $$\widehat{\text{I}\text{N}\text{B}}$$. This regression framework allows adjusting for potential confounders and moderators [9, 10]. At WTP = $0/AFD, the $$\widehat{{\text{n}\text{b}}_{\text{i}}}$$ is reduced to $$-\widehat{{\text{C}}_{\text{i}}}$$; $$\widehat{\text{I}\text{N}\text{B}}$$ becomes $$\widehat{-{\Delta }\text{C}}\text{I}\text{n}\text{t}\text{e}\text{r}\text{v}\text{e}\text{n}\text{t}\text{i}\text{o}\text{n}$$. With increasing WTP, there is a greater emphasis on$${\widehat{ \text{E}}}_{\text{i}}$$, which eventually will tend towards infinity, and $$\widehat{\text{I}\text{N}\text{B}}$$ will become $$\widehat{{\Delta }\text{E}}\text{I}\text{n}\text{t}\text{e}\text{r}\text{v}\text{e}\text{n}\text{t}\text{i}\text{o}\text{n}$$.

The new intervention’s adjusted cost-effectiveness probability is the one-sided p-value associated with the hypothesis that $$\widehat{{\beta }}\text{I}\text{n}\text{t}\text{e}\text{r}\text{v}\text{e}\text{n}\text{t}\text{i}\text{o}\text{n}>0$$. It is used to create a CEAC for different WTP thresholds [63]. To do so, a graph was generated by plotting the one-sided p-values on the y-axis, which is the probability of cost-effectiveness, obtained at a range of WTP from $0/AFD to $100/AFD (x-axis). A probability threshold of 95% was used to determine if tCBT+TAU would be cost-effective compared to TAU alone. In Table 1, bilateral p-values are presented.

**Table 1:** Number of comorbid anxiety disorders as the moderator of the cost-effectiveness of tCBT + TAU compared to TAU as shown by multivariable linear regression

	Willingness to pay threshold ($CAN)																									
	WTP (0)			WTP (10)			WTP (20)			WTP (30)			WTP (40)			WTP (50)				WTP (60)			WTP (80)			WTP (100)
	$$\widehat{\beta }$$																									
**tCBT intervention (ref: TAU)** ^**a**^	*-2 199*			*-1 855*			*-1 511*			*-1 167*			*-823*			*-479*				*-135*			*553*			*1 241*
p-value	0.088			0.157			0.265			0.411			0.584			0.765				0.937			0.778			0.579
**Number of comorbid anxiety** **disorders (ref: none)**																										
One	*-663*			*-926*			*-1 188*			*-1 451*			*-1 714*			*-1 977*				*-2 240*			*-2 765*			*-3 291*
p-value	0.553			0.417			0.316			0.245			0.196			0.163				0.140			0.113			0.099
Two	*-1 430*			*-1 711*			*-1 991*			*-2 271*			*-2 552*			*-2 832*				*-3 112*			*-3 673*			*-4 234*
p-value	0.310			0.233			0.179			0.143			0.120			0.105				0.095			0.084			0.080
Three	*-4 964*			*-5 684*			*-6 403*			*-7 123*			*-7 842*			*-8 562*				*-9 282*			*-10 721*			*-12 160*
p-value	0.015			0.007			0.003			0.002			0.001			0.001				0.001			0.001			0.001
Intervention * nb of comorbid anxiety disorders (ref: TAU, none) ^**a**^																										
tCBT + TAU * One	*895*			*1 123*			*1 352*			*1 580*			*1 809*			*2 038*				*2 266*			*2 723*			*3 181*
p-value	0.586			0.500			0.432			0.380			0.342			0.315				0.295			0.272			0.261
tCBT + TAU * Two	*2 885*			*2 961*			*3 038*			*3 115*			*3 191*			*3 268*				*3 344*			*3 497*			*3 650*
p-value	0.143			0.140			0.143			0.152			0.165			0.181				0.200			0.241			0.283
tCBT + TAU * Three	*6 171*			*6 295*			*6 418*			*6 542*			*6 665*			*6 789*				*6 912*			*7 159*			*7 406*
p-value	0.016			0.016			0.018			0.022			0.027			0.035				0.044			0.068			0.098

Note. ref = Reference category; TAU = Treatment as usual; tCBT = Transdiagnostic cognitive behavioural therapy; WTP = Willingness to pay threshold. Adjusted for SAD as a principal diagnosis and comorbid PD.

^a^ The beta coefficients represent the incremental net-benefit.

#### Confounders and moderators

First, a series of linear regression analyses were conducted to identify potential confounders, and moderators using the nb_i_ as an outcome. All models included a potential confounder or a moderator, and the intervention group as independent variables. Significant confounders or moderators were added simultaneously to the multivariable models. To be considered a confounder, a variable had to: (1) differ statistically between the two conditions (p-value of the pooled $\widehat{\beta }$<0.05) [64]; (2) cause a 10% change in the beta estimate of the relationship between the intervention group and the outcome when added to the univariate model [65]; as well as (3) be associated (p-value of the pooled $\widehat{\beta }$<0.05) with the outcome [64]. As for moderation analysis, the following basic model was used: $\widehat{{\text{n}\text{b}}_{\text{i}}}=\widehat{{{\beta }}_{1}}\text{I}\text{n}\text{t}\text{e}\text{r}\text{v}\text{e}\text{n}\text{t}\text{i}\text{o}\text{n}+\widehat{{{\beta }}_{2}}{\text{X}}_{2}+\widehat{{{\beta }}_{3}}\left(\text{I}\text{n}\text{t}\text{e}\text{r}\text{v}\text{e}\text{n}\text{t}\text{i}\text{o}\text{n}\text{*}{\text{X}}_{2}\right)+{\alpha }$. If p < 0.05 for $\widehat{{{\beta }}_{3}}$ on at least one WTP threshold tested, ${\text{X}}_{2}$ was considered a potential moderator and was included in the multivariable models [66]. The interaction term between the intervention group and moderating variables, $\widehat{{{\beta }}_{3}}$, were used to represent the INB stratified by category of moderator. A stratified cost-effectiveness acceptability curve was provided for significant moderators.

#### Regression diagnostics and outliers

The twenty datasets obtained by multiple imputation were checked individually [67]. First, standardized residuals vs. standardized predicted value graphs were observed to find abnormal patterns indicating heteroscedasticity across datasets. A Loess curve with Cauchy kernels was fitted in the same graphs to assess the linearity of the relationships between the outcome and independent variables [68]. Skewness and kurtosis parameters were assessed to evaluate the normality of the residuals. Z-values were obtained and compared against a threshold of 3.29 [69, 70]. Variance inflation factors were computed and a threshold of 10 was considered for multicollinearity [71].

Individuals with high leverage (Leverage > 3*p/n [72]; where p equals the number of variables including the constant) were investigated as well as those with absolute DFBETAS equal to or higher than $2\surd \text{n}$ [73]. Cook’s distance was also evaluated with a threshold of 4/n [74]. Data points flagged as outliers or influential observations in five datasets or more were analyzed further to detect any recurring patterns in sociodemographic or clinical characteristics. A plot of standardized residuals vs. Leverage with Cook’s D at 4/n was created to identify influential outliers visually. A threshold of 1 was also considered [75].

In the final adjusted models, regression diagnostic showed a significant deviation from normality when WTP=$0/AFD, as expected due to the skewed nature of cost data ($$\widehat{{\text{n}\text{b}}_{\text{i}}}$$ reduced to $$\widehat{{-\text{C}}_{\text{i}}}$$),for the health system and the limited societal perspectives. Linearity was confirmed by examining the standardized residuals vs. fitted standardized predicted values based on Loess curve with Cauchy kernels. The homoscedasticity postulate was confirmed visually with graphs and using Levene’s test for homogeneity of variances with nb_i_ as an outcome variable and this for both health system and limited societal economic perspectives. Some influential outliers were identified from the standardized residuals against leverage with Cook’s D contour plots. However, no clear pattern was derived from their baseline characteristics, and they were not constant between analyses at WTP=$0/AFD and WTP=$100/AFD. No outlier was removed from analyses, and no multicollinearity was detected in adjusted models. More detail can be found in the Supplementary Results Appendix 2.

An alpha of 0.05 was used to determine statistical significance. IBM SPSS 24 was used to carry out the analysis following the intention-to-treat principle.

## Results

### Baseline participant’s characteristics and missing data

A total of 117 participants were randomized to the tCBT + TAU condition and 114 participants in the TAU condition. At T_1_, respectively, in the tCBT + TAU and TAU conditions, 92 (78.6%) and 95 (83.3%) participants had an available BAI score and data on costs to proceed with the analysis. At T_2_, these numbers dropped to 87 (74.4%) and 90 (78.9%). Missing data were imputed. Since no logical bounds or transformations were applied, we examined the proportions of negative values for total cost after imputation. Across the 20 imputed datasets, from the health system perspective, the proportion of negative total cost data was 2.9%. From the limited societal perspective, this proportion amounted to 0.2%. More details on costs can be found in Chapdelaine et al. (2022) [29].

Participant characteristics have been described elsewhere [32] and in the Supplementary Results Appendix (see Table S1). Briefly, most of the sample consisted of women (85.7%), and the average age was 37 years old. Most participants worked full-time (60.6%) and considered themselves as being in a satisfactory economic situation (76.6%). Most participants had post-secondary education, and one-tenth had a high school degree or less. A third of participants considered themselves in very good or excellent physical health (34.2%). Half of the sample presented a GAD as a principal anxiety disorder (52.8%). Anxiety-anxiety comorbidity was high as three in four participants had at least one other comorbid anxiety disorder, the most frequent being SAD (33.8%). Close to one in four participants also had a comorbid major depressive disorder. Despite randomization, an imbalance remained in the prevalence of specific principal anxiety disorders between groups. In those randomized to tCBT + TAU, as compared to TAU, the proportion with a principal diagnosis of GAD was significantly higher while SAD was lower. There were also more individuals with comorbid PD in the tCBT + TAU group. As different types of anxiety disorders could lead to differences in healthcare utilization patterns [76] and consequently potentially impact healthcare costs as well as symptomatology, these were considered as relevant control variables in the analyses.

### Confounders and moderators associated with the cost-effectiveness of tCBT

**Fig. 1:**
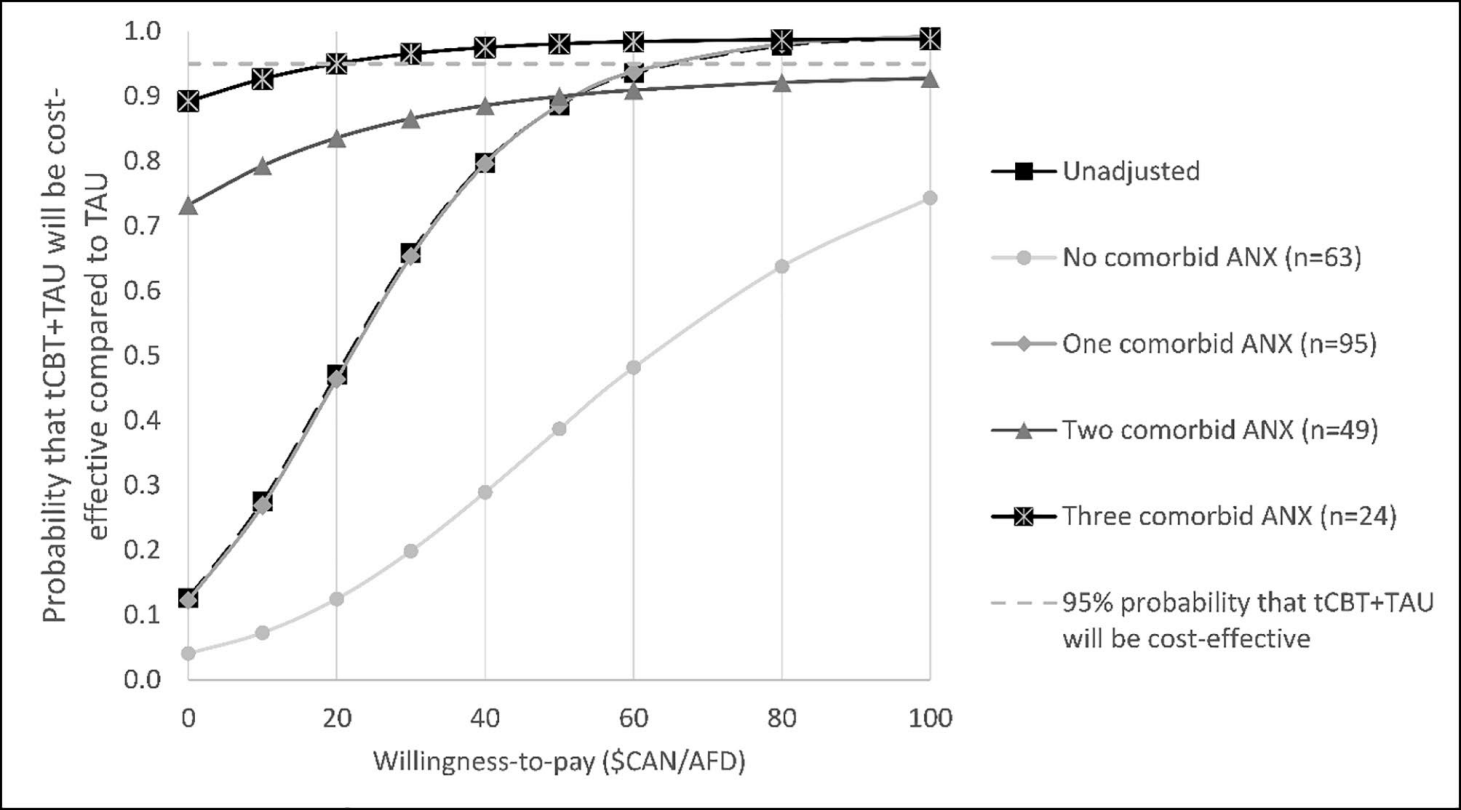
Limited societal perspective cost-effectiveness acceptability curve adjusted and stratified by the number of comorbid anxiety disorder **Note**. Models were stratified by anxiety-anxiety comorbidity with adjustment for having a social anxiety disorder as the principal diagnosis and having a comorbid panic disorder. Considered anxiety disorders are panic disorder, agoraphobia, social anxiety disorder and generalized anxiety disorder. ANX = anxiety disorder; TAU = Treatment as usual; tCBT = transdiagnostic cognitive behavioural therapy.

Table 1 shows the multivariable linear regression analyses of the nb_i_ as a function of the identified confounders and moderators affecting the incremental net-benefit and therefore, the cost-effectiveness of tCBT. From the limited societal perspective, SAD as a principal diagnosis and comorbid PD were identified as confounding variables. There was a significant interaction between the intervention group and the number of comorbid anxiety disorders. It showed that compared to those with no comorbid anxiety disorders, for those with all four included anxiety disorders (GAD, PD, SAD, AGO), tCBT + TAU appeared cost-effective (p < 0.05; WTP≤$60/AFD). The association was significant at a WTP=$0/AFD and indicated that compared to those with no comorbidity, those with three comorbid anxiety disorders in the control group had higher mental health costs than their counterparts in the intervention condition. Figure 1 shows the CEAC stratified by the number of comorbid anxiety disorders. The probability of cost saving increases with the number of anxiety disorders (WTP=$0/AFD). Also, the probability of cost-effectiveness of tCBT + TAU increases with the number of anxiety disorders at WTP≤$50/AFD. At a threshold of $20/AFD, there was a 95% probability that tCBT + TAU would be cost-effective compared to TAU in those with three comorbid anxiety disorders. In those with no anxiety disorder comorbidity, the estimated probability was 12% at the same threshold.

From the health system perspective, comorbid PD and, as principal diagnoses, SAD and GAD, were included in the multivariable model as potential confounders. From the health system perspective, none of the variables studied reached statistical significance as a moderator. The adjusted CEAC showed that at a WTP of $25/AFD, there was ≥ 95% of probability that tCBT + TAU would be cost-effective compared to TAU (See Table S2 and Figure S1 in the Supplementary Results).

## Discussion

Using data from an RCT opposing tCBT + TAU to TAU alone, this study aimed to identify moderators that could significantly affect the cost-effectiveness (i.e., the INB) of tCBT + TAU vs. TAU from the limited societal and health system perspectives. The probability of cost-effectiveness varied according to the number of anxiety comorbidities. tCBT + TAU had a 95% probability of being cost-effective versus TAU at a WTP=$20/AFD in those with three anxiety disorder comorbidities and 12% in those with no anxiety comorbidity. From the health system perspective, none of the interaction terms tested were significant.

The results showed that the number of comorbid anxiety disorders was a significant moderator of the association between the intervention group and the nb_i_ from the limited societal perspective, supporting the use of tCBT in individuals with increasing comorbid anxiety disorders. Individuals in the tCBT + TAU group with more comorbidities had less mental health costs and more AFDs than those in the TAU group on the 8-month time horizon and, therefore, a higher probability of cost-effectiveness. These findings do not suggest that tCBT is not cost-effective for those with no comorbidity; it may be cost-effective but at a higher willingness to pay threshold.

The current findings complement those of Norton et al. (2021), from this same trial [31], which showed that the rate of comorbidity (mainly between anxiety and major depressive disorder) was significantly lower from baseline to post-treatment and 8-month post-treatment in the tCBT + TAU condition compared to TAU, with comorbidity remission being defined as having no or subclinical symptom severity scores at evaluation [77]. The latter results replicated those obtained in a previous efficacy trial on tCBT [78]. Anxiety-anxiety comorbidities represent the norm [79–82] and have been associated with higher rates of chronicity, more severe depressive, anxiety and avoidance symptoms, and more social disability [83]. tCBT could represent a cost-effective approach to alleviate the burden of individuals with comorbidities.

To our knowledge, there is a lack of literature on factors predicting change in health system and societal costs associated with health service utilization after treatment with CBT. The current findings showed an interaction between the intervention and the presence of anxiety disorder comorbidities. This suggests that comorbidities could moderate costs in CBT trials, and this finding will need further exploration.

Some limitations of the study need to be considered. First, it is noteworthy that the RCT on which the present study was based was powered for the primary outcome, which was the BAI score, and not cost. A lack of power can have a more severe impact when interpreting the linear regression models at WTP=$0/AFD (i.e., ΔC), because of the skewed nature of costs. Second, although regression diagnostics were done to assess the correctness of our models, they were not validated with data-splitting to ensure its adequacy as the sample was limited. Results will need to be replicated. Third, although not extreme, regression diagnostic highlighted skewness in data, particularly at low WTP when costs have a more considerable impact on the nb_i_ distribution. A nonparametric method to obtain the p-values for the CEAC is bootstrapping. However, in addition to being able to adjust models, as underlined by Hoch et al., (2006), the net-benefit regression framework has the advantage of computing: (1) the INB, (2) the mean nb_i_ of the control group (β_0_), the mean nb_i_ of the experimental group (β_0_ + β_1_) as well as regression information (residuals, R^2^, etc.) [84]. Fourth, more than one individual in three had missing clinical or cost data at the 4-month post-treatment assessment, introducing a potential bias. The multiple imputation model included variables significantly associated with missingness and baseline variables, minimizing the effect of attrition on results. As no logical bounds or transformations were applied in the multiple imputation processes to prevent possibly biasing findings [61, 85, 86], we examined the proportions of negative values of total cost after multiple imputation and these were minimal. Fifth, to this day, there is no recommendation as to the value of an anxiety-free day. The interaction between the intervention and the number of anxiety comorbidities was statistically significant at willingness to pay thresholds up to CAN $60/AFD. That being said, the importance of this finding will depend on the future societal value of an AFD. Sixth, due to the high number of statistical analyses, there is an increased risk for type I errors, but no correction was made due to this study’s exploratory nature. Finally, AFD represents a measure of the variation of anxiety symptoms over time. As quality-adjusted life years is a standard outcome in economic evaluation, representing both quantity and quality of life, future studies could assess the moderators of its association with tCBT on a longer time horizon with a measure responsive to change in a mental health context.

As for the generalizability of results, the following need to be considered. This study is a secondary data analysis from an RCT, carried out in a French-speaking Canadian province, for which participants were mostly white females self-reporting a favorable socioeconomic status. Future research on potential moderators affecting the cost-effectiveness of tCBT should consider a naturalistic study design factoring in contextual elements such as access to mental health care and including a more diverse sample. Also, the recruitment process was designed to represent the primary care sector in a public health system. It limits the generalization of results to other specialty and health system contexts. Moreover, the public healthcare context is to be considered when analyzing costs related to service use as most medical care and treatments are covered or partially covered, and insurance coverage may affect help-seeking behaviours [87].

## Conclusion

The current study findings showed that increased anxiety disorder comorbidity influenced the cost-effectiveness of tCBT + TAU vs. TAU from a limited societal perspective. Evaluating the cost-effectiveness of tCBT added to treatment as usual in populations with different anxiety disorder comorbidities and settings is needed to improve timely access to quality mental health resources.

## Electronic supplementary material

Below is the link to the electronic supplementary material.

Supplementary Material 1: Supplementary methods appendix

Supplementary Material 2: Supplementary results appendix

## Data Availability

The datasets generated and/or analysed during the current study are not publicly available due the multicentre ethical approval and the consent forms that do not allow for open access to data, but are available from the corresponding author on reasonable request.
