# Supplementary material for: Moderators of the cost-effectiveness of transdiagnostic CBT for anxiety disorders over an 8-month time horizon using a net-benefit regression framework
Source: BMC Health Serv Res. 2023 Jun 8;23:596. doi: 10.1186/s12913-023-09468-7 (PMC10251685; doi:10.1186/s12913-023-09468-7)
Supplement: Supplementary file 1 — Supplementary Material 1: Supplementary methods appendix [file 12913_2023_9468_MOESM1_ESM.docx]

# **Costing supplement**

**Table 1**

Description of mental health costs and valuation

| Health system (HS) cost | | | |
| --- | --- | --- | --- |
| Cost item | Unit | Valuation | Data source |
| Costs for mental health-related medications for those covered by the public drug plan^a^ | Pill taken | Name, dosage, start-date | [1] |
| Health system mental health care visits^b,c^ |  |  |  |
| *Outpatient* | Visit | Type of professional; including professional’s fees | [2–5] |
| *Emergency* | Visit | Including professional’s fees | [2–5] |
| *Hospitalization* | Stay | General scenario: A standard hospitalization scenario was applied including physician consultation fees [6] | [2–5] |
| **Limited societal perspective** | | | |
| Direct medical cost | | | |
| All HS costs |  |  |  |
| Costs for mental health-related medication^a,d^ | Pill taken | Name, dosage, start-date | [1]^e^ |
| Mental health care visits outside the public healthcare system | Visit | Type of professional; | Professional association, estimations from random practice in each region |
| Costs of complementary and alternative medicine | Monthly cost | - | Self-reported |
| Indirect medical cost | | | |
| Time for mental health care appointments^f^ | Visit | - 4 hours for an outpatient visit (and tCBT session)  - 8 hours for an emergency department visit  - 24 hours per day for hospitalization  [7–9] | Self-reported number of visits |
| Cost of transportation for mental health care visits and the intervention | Visit; roundtrip | - Mean of transportation (e.g., taxi, metro, car, walking).  - City’s diameter | [10–17] |
| Need for assistance in day-to-day activities because of mental health | Hours per week | Opportunity cost defined as the minimum wage | [18] |
| Indirect cost | | | |
| Long term medical leave (absenteeism) | Week | Self-reported. Average wage in Quebec by sex and age^g^. Adjusted for the friction period [19]. | [18, 20] |
| Presenteeism | Hour | Difference between work output when in a fully productive state (100%) and self-reported productivity on a scale of 1 to 10 on the previous 28 days – Absolute presenteeism [21]  Average wage in Quebec by sex and age^g^ | [18, 20] |
| **Intervention cost** | | | |
| Therapist time in session | Hour; self-reported | Private therapist: reported fees  Public therapist: Wage based on the average experience | [22] |
| Preparation time for sessions | Hour; self-reported | Private therapist: reported fees  Public therapist: Wage based on the average experience | [22] |
| Individual pre-therapy appointment | Hour; based on the average length | Private therapist: reported fees  Public therapist: Wage based on the average experience | [22] |
| Telephone follow-up to enhance compliance and minimize loss to follow-up | Hour; self-reported | Private therapist: reported fees  Public therapist: Wage based on the average experience | [22] |
| Participant booklet | Items | Impression fees |  |
| General overhead costs | Hour or m^3 h^ | Based on activity centers: facility operations [electricity, insurance, heating, ventilation, air-conditioning, water], security, hygiene and cleanliness – operational tasks and finally, secretariat [based on average hourly rate]). | [2] |
| ^a^ Including wholesaler mark up and pharmacist fees [1]  ^b^Including furniture, social benefits, staff salary, doctor salary  ^c^ With a marginal mark-up for overhead [23] and opportunity and building-depreciation costs [24]  ^d^ With adjusted pharmacist fees for those on a private insurance plan [25]  ^e^ For the cost of medications not covered by the RGAM, price was given by the Association québécoise des pharmaciens propriétaires [25]  ^f^ Cost adjusted by the weight of the number of expected hours worked in a week  ^g^ Estimated using the friction cost method based on periods corresponding to the average duration of unemployment [19, 26] adjusted for age and sex [19], with a factor of elasticity of 0.80 [27]  ^h^ Estimated for a 5mx5mx2,4m room | | | |

# **References**

1. Régie de l’assurance maladie du Québec. Liste des médicaments [List of medications]. 2018. https://www.ramq.gouv.qc.ca/fr/media/6056. Accessed 27 Jul 2020.

2. Ministère de la Santé et des Services sociaux. AS-471 – RAPPORTS FINANCIERS ANNUELS DES ÉTABLISSEMENTS [AS-471 - ANNUAL FINANCIAL REPORTS OF INSTITUTIONS]. 2017.

3. Ministère de la Santé et des Services sociaux. Rapports statistiques annuels des CH, CHSLD et CLSC 2016-2017 [Annual statistical reports of the CH, CHSLD and CLSC 2016-2017]. 2017.

4. Régie de l’assurance maladie du Québec. Manuel des médecins omnipraticiens (n 100) [General practitioners’ manual (n 100)]. 2016. https://www.ramq.gouv.qc.ca/SiteCollectionDocuments/professionnels/manuels/100-facturation-omnipraticiens/000_complet_acte_omni.pdf. Accessed 27 Jul 2020.

5. Régie de l’assurance maladie du Québec. Manuel des médecins spécialistes (MAJ89) [Specialist physicians’ manual (MAJ89)]. 2016;:506. http://collections.banq.qc.ca/ark:/52327/bs2580637. Accessed 27 Jul 2020.

6. Vasiliadis H-M, Dionne P-A, Préville M, Gentil L, Berbiche D, Latimer E. The excess healthcare costs associated with depression and anxiety in elderly living in the community. The American Journal of Geriatric Psychiatry. 2013;21:536–48.

7. Vasiliadis H-M, Marchand J-S, Gosselin S, Guerra S, Breton M. Emergency Deparments Performance and Length of Stays Stagnation: A 24/7 Public Service Working from Nine-to-Five? In: Conference: 2019 CAHSPR Scientific Conference. Halifax, Canada. 2019.

8. Vasiliadis H-M, Latimer E, Dionne PA, Préville M. The costs associated with antidepressant use in depression and anxiety in community-living older adults. Canadian Journal of Psychiatry. 2013;58:201–9.

9. Lamoureux-Lamarche C, Vasiliadis H-M, Préville M, Berbiche D. Healthcare use and costs associated with post-traumatic stress syndrome in a community sample of older adults: Results from the ESA-Services study. Int Psychogeriatr. 2016;28:903–11.

10. Commission des transports du Québec. Mécanisme de fixation des tarifs de taxi. 2017. https://www.ctq.gouv.qc.ca/transport-remunere-de-personnes-par-automobile/tarifs-de-transport-remunere-de-personnes-par-automobile/mecanisme-de-fixation-des-tarifs-de-taxi/. Accessed 6 Oct 2017.

11. Statistics Canada. Québec, Quebec (Code 2423) and Canada (Code 01) (table). Census Profile. 2011 Census. 2012. http://www12.statcan.gc.ca/census-recensement/2011/dp-pd/prof/index.cfm?Lang=E. Accessed 21 Nov 2017.

12. Statistics Canada. Sherbrooke, Quebec (Code 2443027) and Quebec (Code 24) (table). Census Profile. 2011 Census. 2012. http://www12.statcan.gc.ca/census-recensement/2011/dp-pd/prof/index.cfm?Lang=E. Accessed 21 Nov 2017.

13. Statistics Canada. Laval, Quebec (Code 2465) and Canada (Code 01) (table). Census Profile. 2011 Census. 2012. http://www12.statcan.gc.ca/census-recensement/2011/dp-pd/prof/index.cfm?Lang=E. Accessed 21 Nov 2017.

14. Société des transports de Laval. Tarifs et points de vente. 2017. https://stlaval.ca/tarifs/grille. Accessed 6 Oct 2017.

15. Société de transport de Sherbrooke. Tarifs. 2017. https://www.sts.qc.ca/307-Tarifs.html. Accessed 6 Oct 2017.

16. Réseau de transport de la Capital. Grille des tarifs. 2017. https://www.rtcquebec.ca/tarifs-et-achat/tarifs-et-titres/grille-des-tarifs. Accessed 6 Oct 2021.

17. Gouvernement du Canada. Taux des allocations pour frais d’automobile. 2017. https://www.canada.ca/fr/agence-revenu/services/impot/entreprises/sujets/retenues-paie/avantages-allocations/automobile/allocations-frais-automobile-vehicule-a-moteur/taux-allocations-frais-automobile.html. Accessed 6 Oct 2021.

18. Commission des normes de l’équité de la santé et de la sécurité du travail. Historique du salaire minimum [Minimum wage review]. 2017. https://www.cnt.gouv.qc.ca/salaire-paie-et-travail/salaire/historique-du-salaire-minimum/. Accessed 17 Aug 2020.

19. Statistics Canada. Table 282-0048 - Labour force survey estimates (LFS), duration of unemployment by sex and age group, annual (persons unless otherwise noted). CANSIM (database). 2017. http://www5.statcan.gc.ca/cansim/a47. Accessed 22 Aug 2017.

20. Institut de la statistique du Québec. Rémunération horaire moyenne des employés, résultats selon le sexe pour diverses caractéristiques de la main-d’oeuvre de l’emploi et du milieu de travail, Québec, Ontario et Canada. [Average hourly earnings of employees, results by gender for various...]. Statistique Canada, Enquête sur la population active, 2016, adapté par l’Institut de la statistique du Québec (ISQ). 2017. http://www.bdso.gouv.qc.ca/pls/ken/ken213_afich_tabl.page_tabl?p_iden_tran=REPERLFBVLS1998126029709Zv9)J&p_lang=1&p_m_o=ISQ&p_id_ss_domn=1096&p_id_raprt=2769. Accessed 8 Nov 2017.

21. Kessler R, Barber C, Beck A, Berglund P, Cleary PD, McKenas D, et al. The World Health Organization Health and Work Performance Questionnaire (HPQ). J Occup Environ Med. 2003;45:156–74.

22. Comité patronal de négociation du secteur de la santé et des services sociaux. 1546 - Psychologue. 2017. http://n02.pub.msss.rtss.qc.ca/AfficherDetails.aspx?TitreEmploi=1546. Accessed 6 Oct 2021.

23. Tan SS, Van Ineveld BM, Redekop WK, Hakkaart-Van Roijen L. Comparing methodologies for the allocation of overhead and capital costs to hospital services. Value in Health. 2009;12:530–5.

24. Rosenheck RA, Frisman LK, Neale MS. Estimating the capital component of mental health care costs in the public sector. Administration and Policy in Mental Health and Mental Health Services Research. 1994;21:493–509.

25. Association québecoise des pharmaciens propriétaires. Article de la presse sur les honoraires des pharmaciens : L’AQPP rétablit certains faits. 2018. https://www.monpharmacien.ca/en/medias/communiques-de-presse/article-de-la-presse-sur-les-honoraires-des-pharmaciens-laqpp-retablit-certains-faits/. Accessed 14 Apr 2020.

26. Hopkins R, Goeree R, Longo C. Estimating the national wage loss from cancer in Canada. Current Oncology. 2010;17:40–9.

27. Koopmanschap MA, Rutten FF, van Ineveld MB, van Roijen L. The friction cost method for measuring indirect costs of disease. J Health Econ. 1995;14:171–89.
