# Supplementary material for: Moderators of the cost-effectiveness of transdiagnostic CBT for anxiety disorders over an 8-month time horizon using a net-benefit regression framework
Source: BMC Health Serv Res. 2023 Jun 8;23:596. doi: 10.1186/s12913-023-09468-7 (PMC10251685; doi:10.1186/s12913-023-09468-7)
Supplement: Supplementary file 2 — Supplementary Material 2: Supplementary results appendix [file 12913_2023_9468_MOESM2_ESM.docx]

**Table S1**

Baseline characteristics of the sample

|  | tCBT+TAU (n=117) | TAU (n=114) |
| --- | --- | --- |
| Baseline characteristic | | |
| Sociodemographic | | |
| Age (years), mean ± S.D. | 37.8 ± 12.2 | 36.2 ± 11.8 |
| Sex (women) | 101 (86.3) | 97 (85.1) |
| Education |  | |
| *High school or less* | 9 (7.8) | 14 (12.3) |
| *Collegial or vocational* | 57 (49.1) | 53 (46.5) |
| *University* | 51 (43.6) | 47 (41.2) |
| Marital status |  | |
| *Single, separated or widowed* | 51 (43.6) | 54 (47.8) |
| *In a relationship* | 66 (56.4) | 59 (52.2) |
| Occupation in the last month |  | |
| *Full time* | 73 (62.4) | 67 (58.8) |
| *Part time* | 20 (17.1) | 24 (21.1) |
| *Non remunerated* | 18 (15.4) | 18 (15.8) |
| *Leave of absence due to mental health reasons* | 6 (5.1) | 5 (4.4) |
| Self-perceived economical situation |  | |
| *At ease* | 21(18.1) | 30 (26.5) |
| *Sufficient* | 67 (57.8) | 59 (52.2) |
| *Poor or worse* | 28 (24.1) | 24 (21.2) |
| Has private complementary health insurance (yes) | 78 (66.7) | 73 (64.0) |
| Has private drug insurance (yes) | 95 (81.2) | 85 (74.6) |
| Has a family practitioner (yes) | 105 (89.7) | 102 (89.5) |
| Clinical | | |
| Takes psychotropic medication (yes) | 88 (75.2) | 82 (71.9) |
| Perceived mental health |  | |
| *Excellent/very good* | 7 (6.0) | 9 (7.9) |
| *Good* | 41 (35.0) | 35 (30.7) |
| *Average or less* | 69 (59.0) | 70 (61.4) |
| Perceived physical health |  | |
| *Excellent/very good* | 46 (39.3) | 33 (28.9) |
| *Good* | 44 (37.6) | 51 (44.7) |
| *Average or less* | 27 (23.1) | 30 (26.3) |
| Beck Anxiety Inventory score, mean ± S.D. | 24.5 ± 11.0 | 23.7 ± 11.2 |
| Principal anxiety disorder |  |  |
| *Generalized anxiety disorder* | 72 (61.5) | 50 (43.9) |
| *Social anxiety disorder* | 26 (22.2) | 42 (36.8) |
| *Panic disorder* | 13 (11.1) | 20 (17.5) |
| *Agoraphobia* | 6 (5.1) | 2 (1.8) |
| Has comorbid major depression (yes) | 29 (24.8) | 26 (22.8) |
| Number of comorbid anxiety disorder(s)^a^ |  | |
| *0* | 29 (24.8) | 34 (29.8) |
| *1* | 48 (41.0) | 47 (41.2) |
| *2* | 24 (20.5) | 25 (21.9) |
| *3* | 16 (13.7) | 8 (7.0) |
| Has other ADIS-5 mental health comorbidities (yes)^b^ | 54 (46.2) | 45 (39.5) |
| tCBT = transdiagnostic CBT; TAU = treatment-as-usual; S.D. = standard deviation. Data were presented with frequency (percentage) unless otherwise indicated.  ^a^ Among those included in the study (generalized anxiety disorder, social anxiety disorder, panic disorder, agoraphobia)  ^b^Among those with at least one count in the current sample: dysthymia, specific phobia, post-traumatic stress disorder, obsessive-compulsive disorder or major depression) | | |

## Regression analysis from the health system perspective

**Table S2**

Multivariable linear regression of the confounders of the cost-effectiveness of tCBT+TAU compared to TAU from the health system perspective

|  | Willingness-to-pay threshold ($CAN) | | | | | | | | | | | | | | | | |
| --- | --- | --- | --- | --- | --- | --- | --- | --- | --- | --- | --- | --- | --- | --- | --- | --- | --- |
|  | WTP (0) | | WTP (10) | | WTP (20) | | WTP (30) | | WTP (40) | | WTP (50) | | WTP (60) | | WTP (80) | | WTP (100) |
|  | $\hat{\beta}$ | | | | | | | | | | | | | | | | |
| **tCBT intervention^a^ (ref: TAU)** | *-630* | | *-194* | | *243* | | *679* | | *1 115* | | *1 552* | | *1 988* | | *2 860* | | *3 733* |
| p-value | 0.000 | | 0.142 | | 0.262 | | 0.027 | | 0.005 | | 0.002 | | 0.001 | | 0.000 | | 0.000 |
| **Clinical factors** |  |  | |  | |  | |  | |  | |  | |  | |  | |
| **Having a SAD**^b^  **(ref: no)** | *51* | | *515* | | *979* | | *1 443* | | *1 908* | | *2 372* | | *2 836* | | *3 764* | | *4 693* |
| p-value | 0.629 | | 0.005 | | 0.001 | | 0.001 | | 0.001 | | 0.001 | | 0.001 | | 0.001 | | 0.001 |
| **Having a GAD**^b^  **(ref: no)** | *83* | | *402* | | *721* | | *1 040* | | *1 358* | | *1 677* | | *1 996* | | *2 634* | | *3 271* |
| p-value | 0.371 | | 0.016 | | 0.010 | | 0.010 | | 0.010 | | 0.010 | | 0.010 | | 0.010 | | 0.010 |
| **Having a comorbid PD (ref: no)** | *6* | | *-346* | | *-698* | | *-1 050* | | *-1 402* | | *-1 755* | | *-2 107* | | *-2 811* | | *-3 515* |
| p-value | 0.939 | | 0.013 | | 0.003 | | 0.002 | | 0.001 | | 0.001 | | 0.001 | | 0.001 | | 0.001 |
| Note. GAD = Generalized anxiety disorder; ref = reference category; SAD = Social anxiety disorder; TAU = Treatment as usual; tCBT = transdiagnostic cognitive behavioural therapy; WTP = Willingness-to-pay threshold.  a The beta coefficients represent the incremental net-benefit  ^b^ as principal diagnosis. | | | | | | | | | | | | | | | | | |

**Figure S1**

Unadjusted and adjusted probability that tCBT+TAU will be cost-effective against TAU from the health system perspective

*
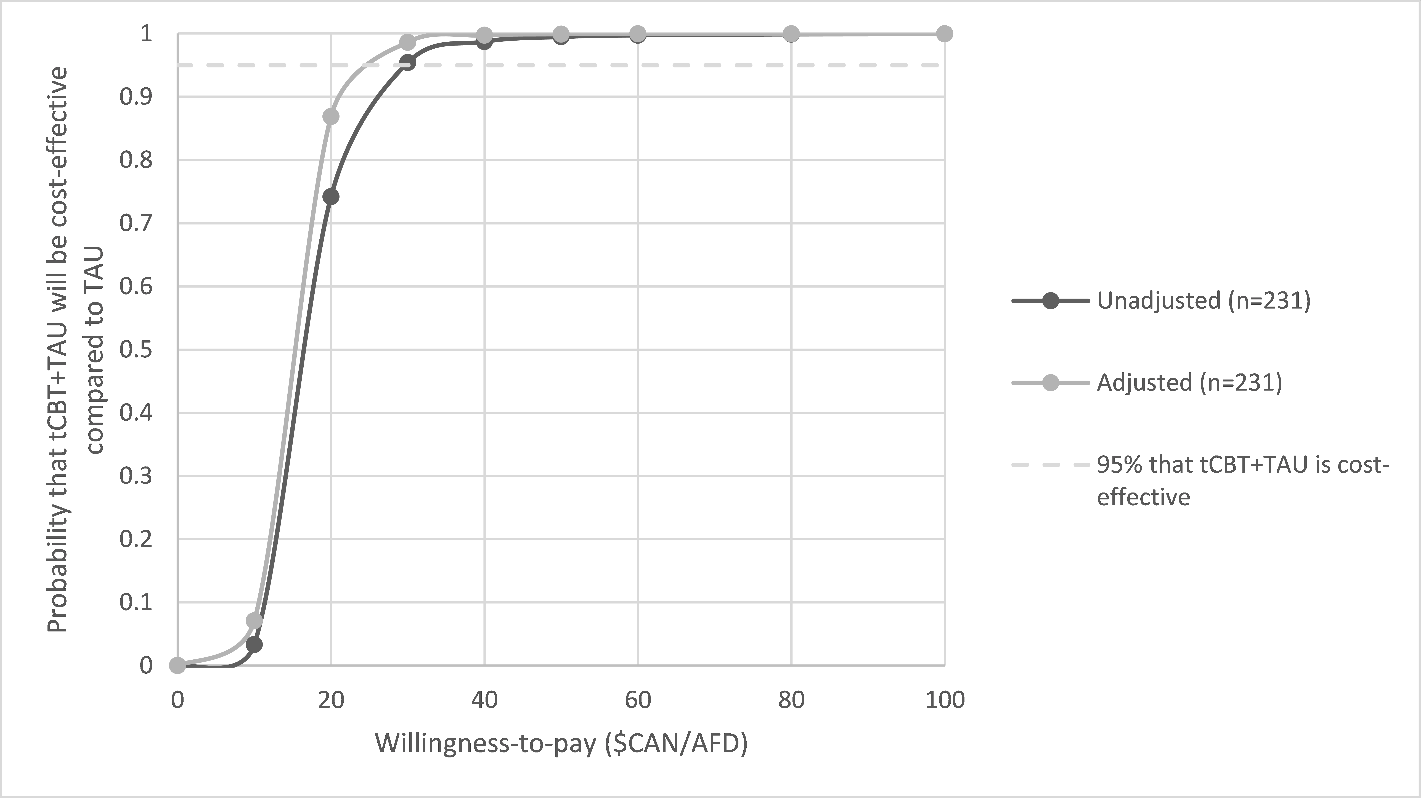
*

Note. Adjusted for confounders: social anxiety disorder as a principal diagnosis, generalized anxiety disorder as a principal diagnosis and the presence a comorbid panic disorder.

AFD = Anxiety-Free Day; TAU = Treatment as usual; tCBT = Transdiagnostic cognitive behavioural therapy

## Regression diagnostics for models based on the limited societal costs

### **Multicollinearity**

The VIF factor indicated no multicollinearity issue.

### **Homogeneity and linearity**

Figure S2 supported a linear model, although, at WTP=$0/AFD, there seemed to be a slight deviation. It also showed no clear pattern of heteroskedasticity, and nonsignificant Levene’s tests confirmed it.

**Figure S2**

Scatterplot of standardized residuals against standardized predicted values with Loess curve with Cauchy Kernels to evaluate homoskedasticity and linearity of data for five imputed datasets from the limited societal perspective

| **WTP=$0/AFD (-ΔC)** | | |
| --- | --- | --- |
| Standardized residuals | 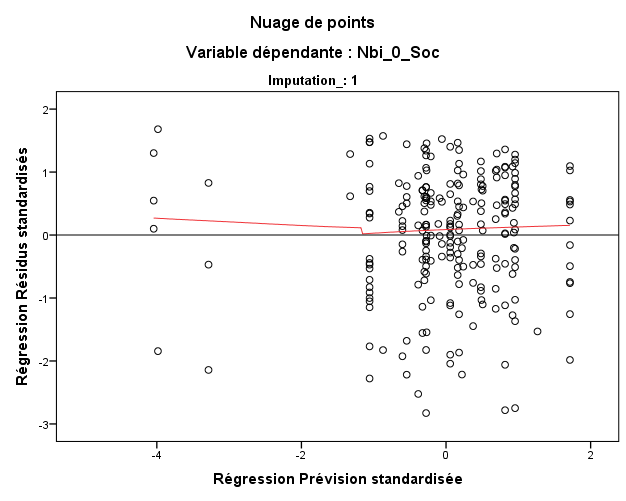 | 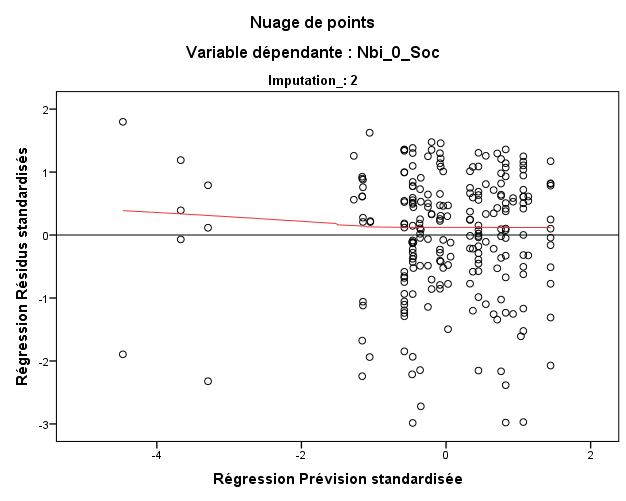 |
|  | 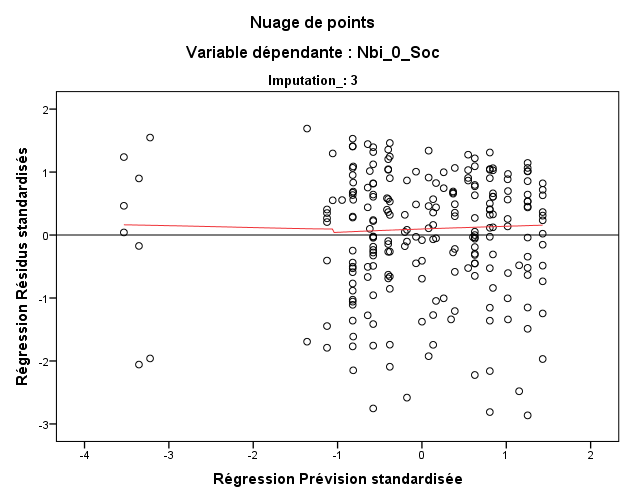 | 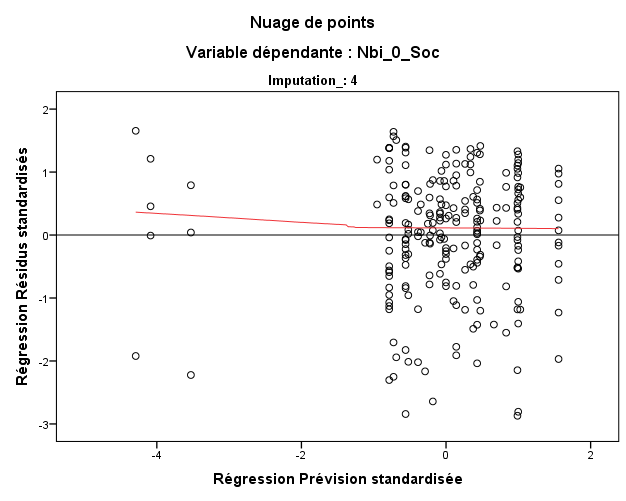 |
|  | Standardized predicted values | |
| Standardized residuals | 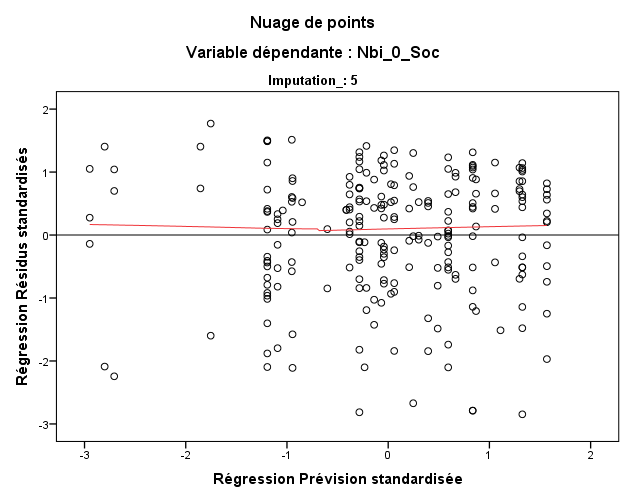 | |
|  | Standardized predicted values | |
| **WTP=$100/AFD** | | |
| Standardized residuals | 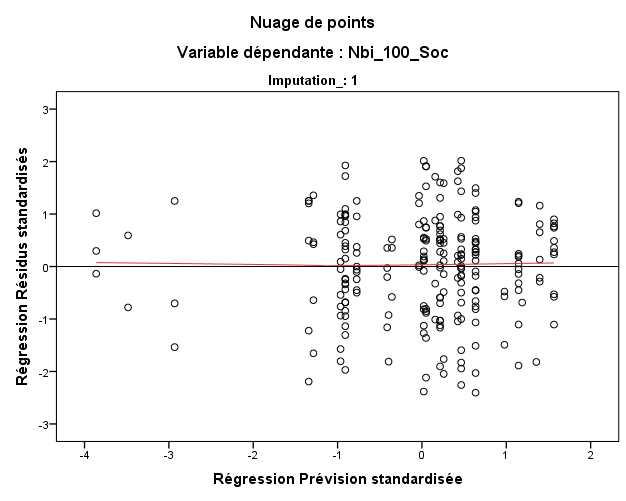 | 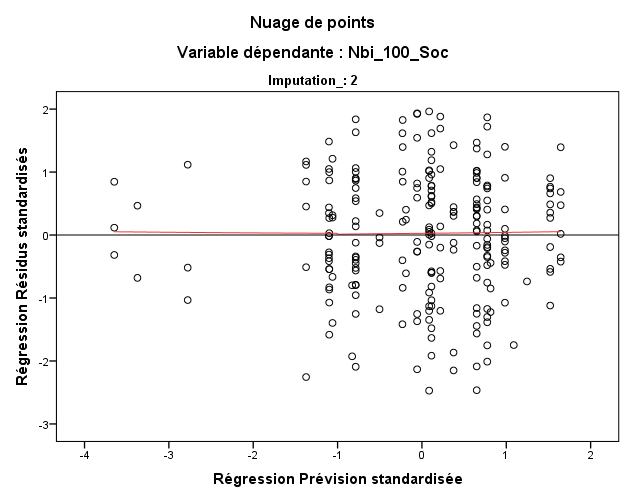 |
|  | 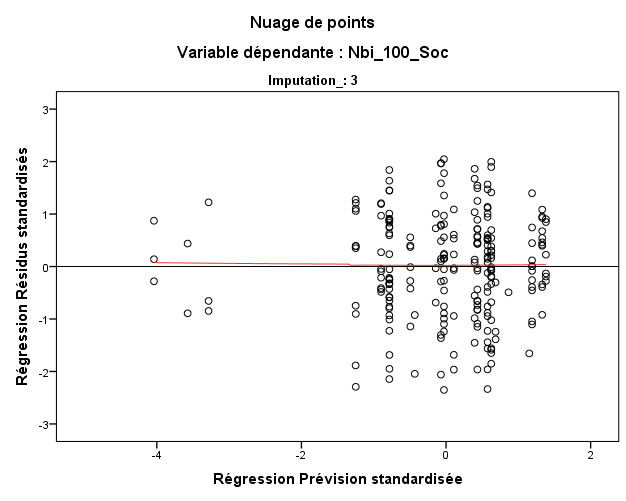 | 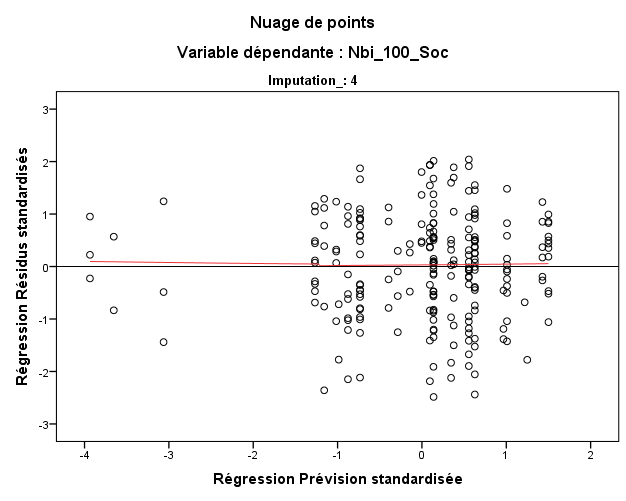 |
|  | Standardized predicted values | |
| Standardized residuals | 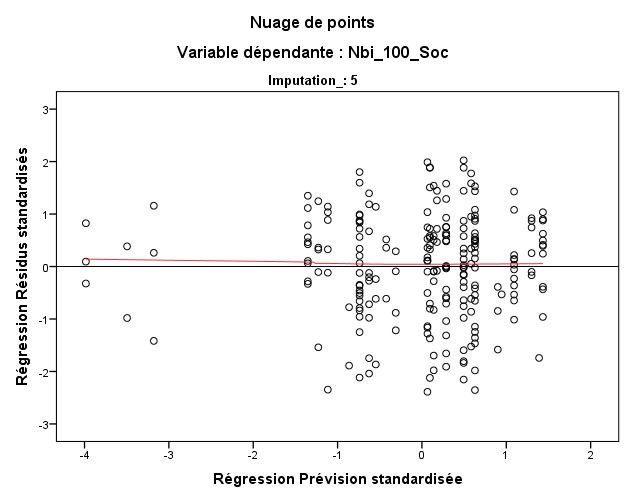 | |
|  | Standardized predicted values | |

Note. All twenty multiply imputed datasets were analyzed. Results from the same five imputed datasets are shown.

### **Skewness and kurtosis**

Skewness and kurtosis values were converted into z-score by dividing by standard error (Table S3) [1]. Due to the relation between standard error and sample size (n=231), a threshold of 3.29 was considered for significant deviation from normality [2]. At WTP=$0/AFD, $\hat{\mathrm{nb}_{i}}$ is reduced to -$\hat{C_{i}}$, and there is a substantial deviation from normality for skewness. The non-normal nature of costs may explain this. Data transformation would make the model harder to interpret. It was deemed better to use data as is.

**Table S3**

Hypothesis testing for skewness and kurtosis of the nb_i_ distribution for five imputed datasets

| Imputation | Skewness (SE=0.160) | Kurtosis (SE=0.319) | S z-value | K z-value |
| --- | --- | --- | --- | --- |
| WTP=$0/AFD | | | | |
| 1 | -0.699 | 0.052 | -4.369 | 0.163 |
| 2 | -0.829 | 0.440 | -5.181 | 1.379 |
| 3 | -0.767 | 0.097 | -4.794 | 0.304 |
| 4 | -0.754 | 0.234 | -4.712 | 0.733 |
| 5 | -0.799 | 0.239 | -5.000 | 0.749 |
| WTP=$100/AFD | | | | |
| 1 | -0.284 | -0.343 | -1.775 | -1.075 |
| 2 | -0.247 | -0.404 | -1.544 | -1.266 |
| 3 | -0.216 | -0.410 | -1.350 | -1.285 |
| 4 | -0.754 | -0.318 | -1.481 | -1.000 |
| 5 | -0.346 | -0.341 | -2.162 | -1.069 |

### **Outliers and individual effect**

#### Leverage

When looking at the leverage of the regression model, five individuals had a value superior or equal to 0.130 (3*p/n; where p equals the number of predictors including the constant). The highest value was 0.150 for an individual in the TAU group at WTP=$0/AFD and 0.143 at WTP=$100/AFD.

#### WTP=$0/AFD

Cook’s D was evaluated to identify potential influential values with a cut-off of 4/n (Cook’D > 0.017). Seventeen participants were identified as having an influential impact across multiple datasets (≥5) on the total sample of 231. Nine were from the TAU condition and eight from the tCBT+TAU condition. The highest value was 0.100. Some say that a threshold of 1 can also be considered to identify potential outliers [3].

Participant's impact on the estimate of the incremental net benefit (INB) was evaluated with the standardised DFBETAS value. Twenty-one individuals appeared more frequently with an absolute value higher than 0.132 ($2/\sqrt{n}$). Thirteen positively impacted the INB (tCBT+TAU: 8, TAU: 5) and eight (tCBT+TAU: 5, TAU:3) decreased it. The absolute highest was 0.388 for an individual in the control group with high societal costs, a below than average number of BAI, and no comorbidity.

The plot of Standardized residuals vs. Leverage with Cook's D contour at 4/n was created to identify influential outliers visually, and an alternative threshold of 1 was also considered (Figure S3). It shows some influential outliers when considering a threshold of 4/n, but none when using a threshold of 1.

**Figure S3**

Standardized residuals against Leverage plot with Cook’s D to detect influential outliers at WTP=$0/AFD from the limited societal perspective


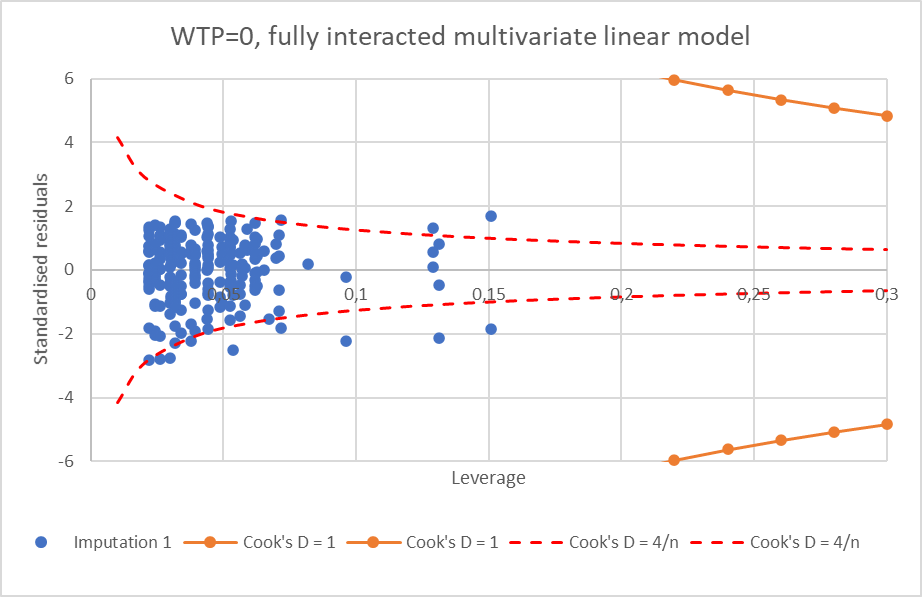


Note. Graph for only one imputation dataset shown. Graphs were similar across datasets.

#### WTP=$100/AFD

Sixteen participants (tCBT+TAU: 9, TAU: 7) were identified as having an influential impact on the regression according to their Cook’s D value, and of those, nine were also influential at WTP=$0/AFD. The highest value was 0.078.

As for participants’ individual impact on the estimate of the incremental net-benefit, no individual had an absolute beta higher than 0.132, and the absolute highest value was 0.070.

The plot of standardized residuals vs. Leverage with Cook's D contour at 4/n shows some influential outliers, but none when using a threshold of one (Figure S4).

**Figure S4**

Standardized residuals against Leverage plot with Cook’s D to detect influential outliers at WTP=$100/AFD from the limited societal perspective


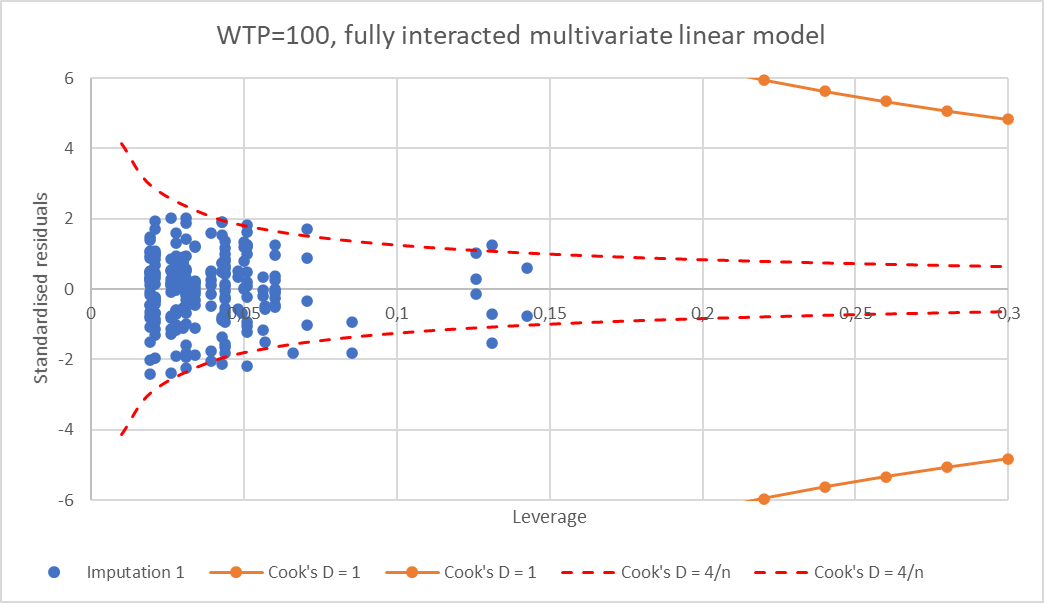


Note. Graph for only one imputation dataset shown. Graphs are similar across datasets.

## Regression diagnostics for models based on the health system costs

### **Multicollinearity**

The VIF factor indicated no multicollinearity issue.

### **Homogeneity and linearity**

Figure S5 supported a linear model and showed no clear pattern indicating heteroscedasticity in the residuals. Levene’s tests were nonsignificant on all datasets at WTP=$0/AFD and WTP=$100/AFD.

**Figure S5**

Scatterplot of standardized residuals against standardized predicted values with Loess curve with Cauchy Kernels to evaluate homoskedasticity and linearity of data for five imputed datasets from the health system perspective

| Fully interacted multiple linear regression | | |
| --- | --- | --- |
| **WTP=$0/AFD (-ΔC)** | | |
| Standardized residuals | 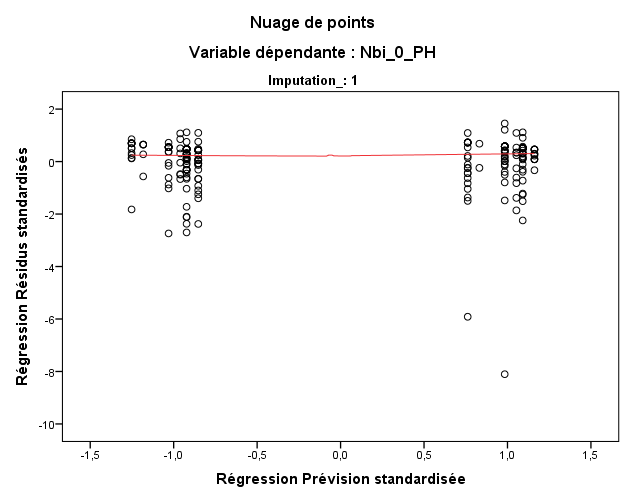 | 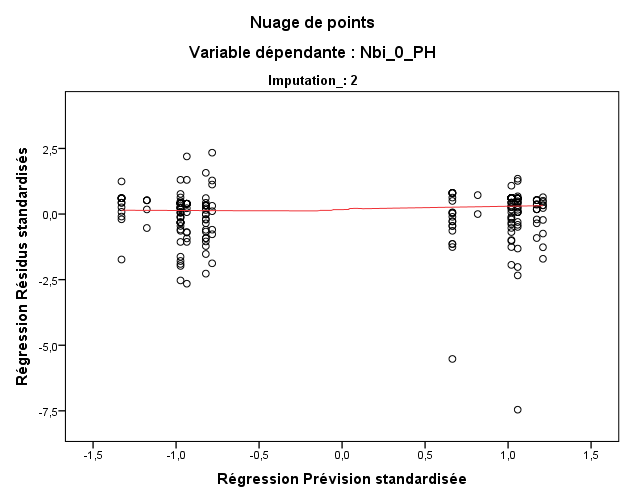 |
|  | Standardized predicted values | |
| Standardized residuals | 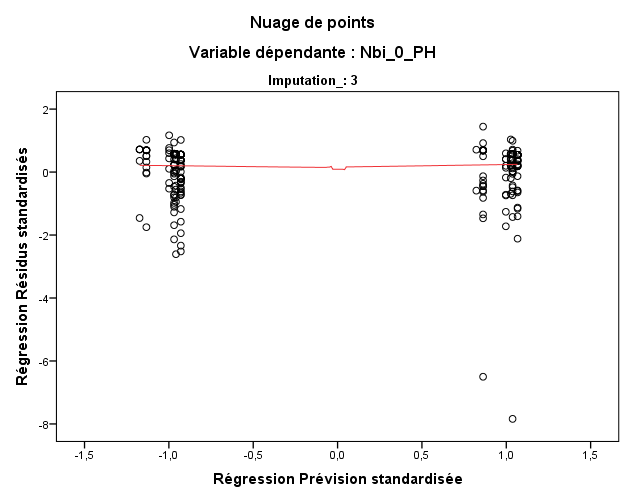 | 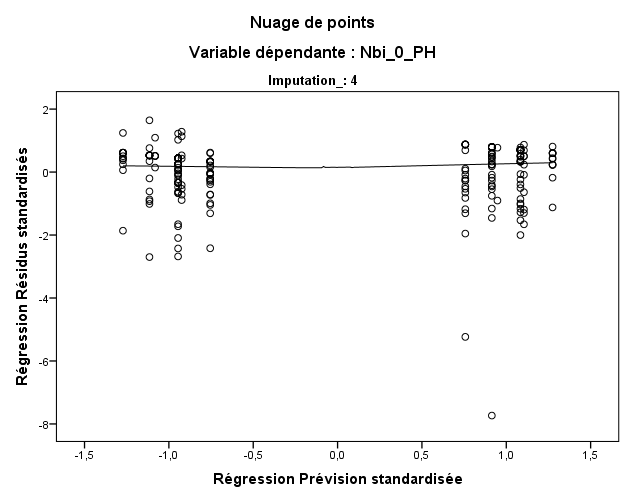 |
|  | Standardized predicted values | |
| Standardized residuals | 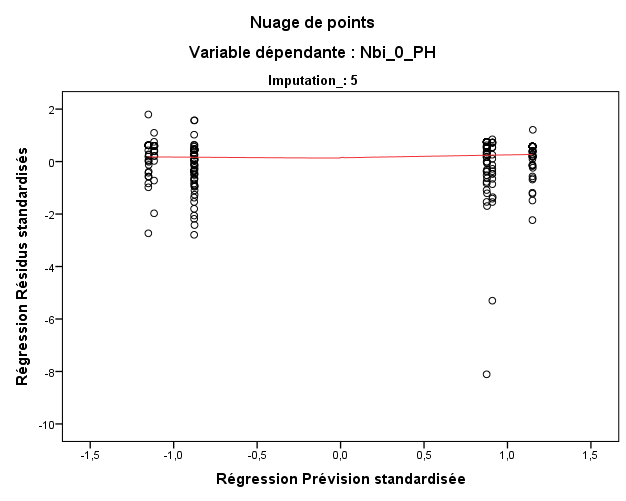 | |
|  | Standardized predicted values | |
| **WTP=$100/AFD** | | |
| Standardized residuals | 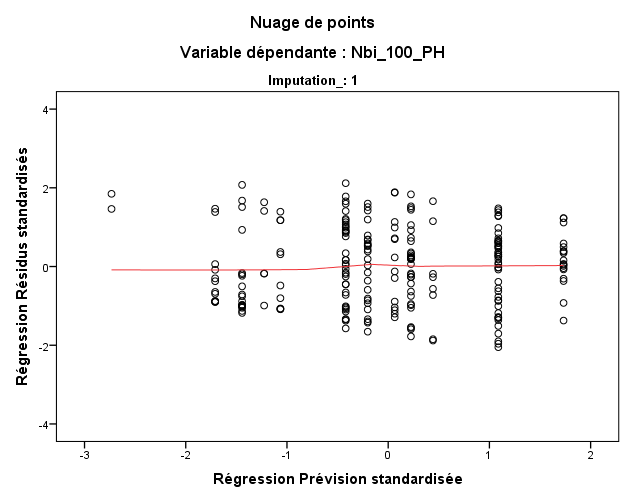 | 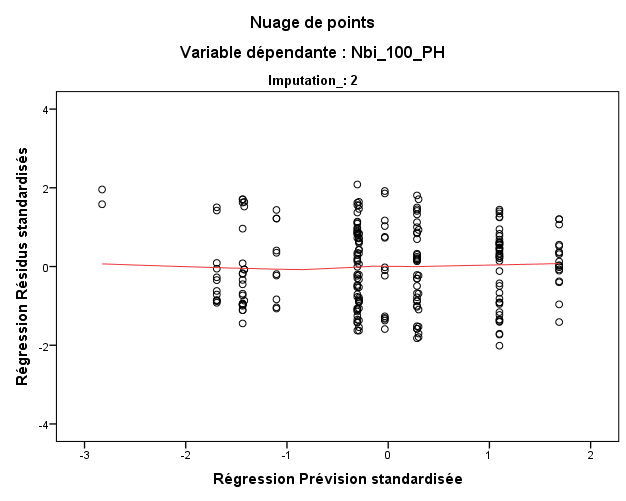 |
|  | Standardized predicted values | |
| Standardized residuals | 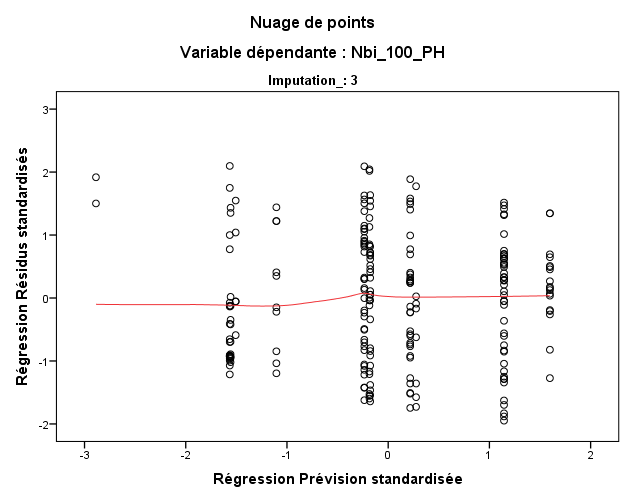 | 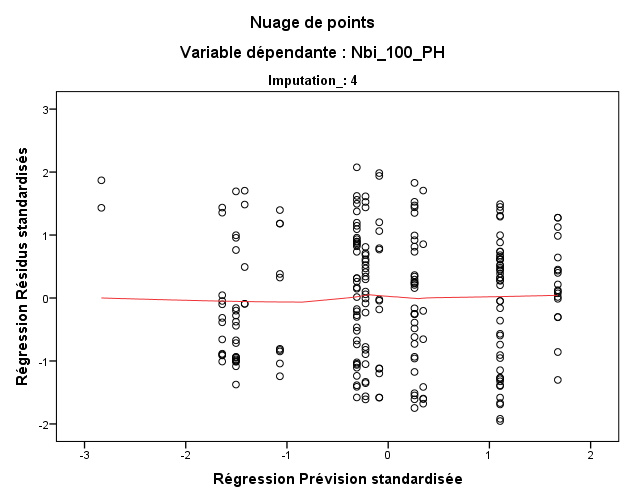 |
|  | Standardized predicted values | |
| Standardized residuals | 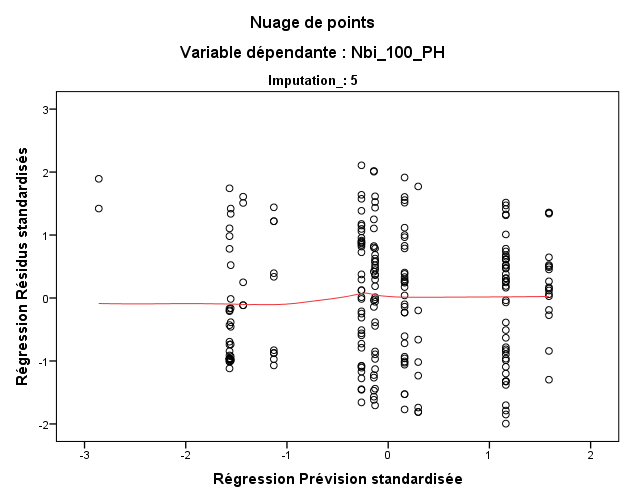 | |
|  | Standardized predicted values | |

### **Skewness and kurtosis**

Two outliers were removed from the analysis so results would be more representative of the distribution. It is possible to observe excess skewness at WTP=$0/AFD, as expected, and kurtosis at WTP=$100/AFD (Table S4).

**Table S4**

Hypothesis testing for skewness and kurtosis of the nb_i_ distribution for five imputed datasets

| Imputation | Skewness (SE=0.161) | Kurtosis (SE=0.320) | S z-value | K z-value |
| --- | --- | --- | --- | --- |
| WTP=$0/AFD | | | | |
| 1 | -0.698 | 0.675 | -4.335 | 2.109 |
| 2 | -0.673 | 0.425 | -4.180 | 1.328 |
| 3 | -0.644 | 0.282 | -4.000 | 0.881 |
| 4 | -0.689 | 0.705 | -4.280 | 2.203 |
| 5 | -0.697 | 0.532 | -4.329 | 1.663 |
| WTP=$100/AFD | | | | |
| 1 | -0.043 | -1.191 | -0.267 | -3.722 |
| 2 | -0.053 | -1.243 | -0.329 | -3.884 |
| 3 | -0.020 | -1.145 | -0.124 | -3.578 |
| 4 | -0.018 | -1.186 | -0.112 | -3.706 |
| 5 | -0.023 | -1.118 | -0.143 | -3.494 |

### **Outliers and individual effect**

#### Leverage

No individual had a leverage value superior or equal to 0.065 (3*p/n), the highest value being 0.041.

#### WTP=$0/AFD

Seven individuals were identified as potentially influential using the Cook's D threshold of 4/n (0.017). Three were from the TAU condition and four from the tCBT+TAU condition.

Participants' individual impact on the estimate of the incremental net benefit was evaluated with the DFBETAS value. Nine individuals appeared more frequently as having an absolute beta higher than 0.132 ($2/\sqrt{n}$); four individuals increased the INB (tCBT+TAU: 1, TAU: 3), and five (tCBT+TAU: 5, TAU: 0) decreased it. From these individuals, five also had substantial influence. No obvious pattern linked these individuals.

The plot of Standardized residuals vs. Leverage with Cook's D contour at 4/n allowed the visual identification of two influential outliers (Figure S6). It shows no potential outliers with influence using a threshold of 1.

**Figure S6**

Standardized residuals against Leverage plot with Cook’s D to detect influential outliers at WTP=$0/AFD from the health system perspective


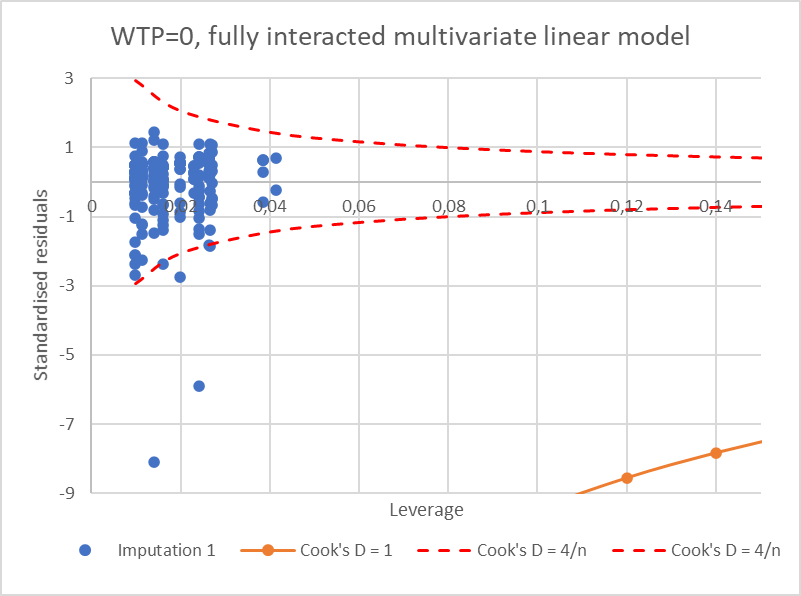


Note. Graph for only one imputation dataset shown. Graphs are similar across datasets.

#### WTP=$100/AFD

Eleven individuals had a high Cook’s D value (seven from the tCBT+TAU group), and five (two from the tCBT+TAU group) had a large individual impact on the incremental net benefit. Two increased the INB (tCBT+TAU: 2) and three (TAU: 3) decreased it. From those who had high Cook’s D value, three also a significant individual impact. Figure S7 shows some individuals that could be influential outliers, and it shows no potential outliers with influence using a threshold of 1.

**Figure S7**

Standardized residuals against Leverage plot with Cook’s D to detect influential outliers at WTP=$100/AFD from the health system perspective


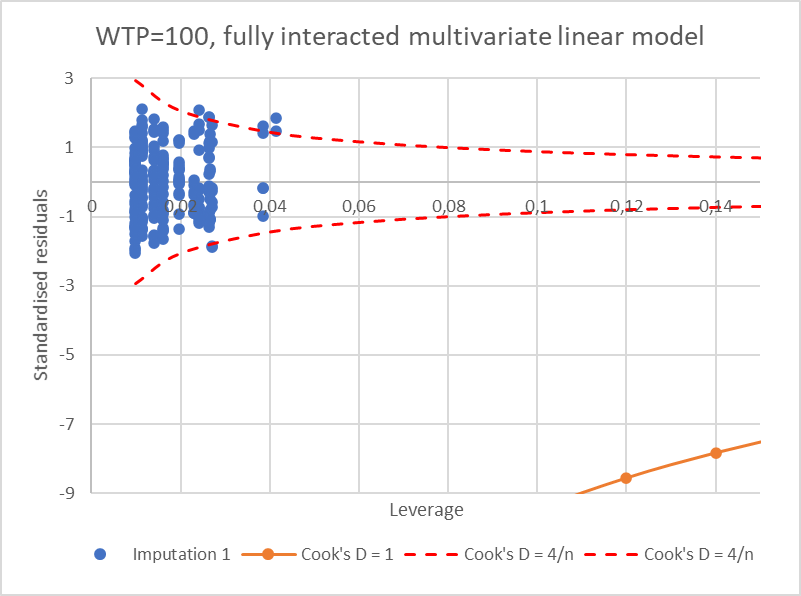


Note. Graph for only one imputation dataset shown. Graphs are similar across datasets.

**References**

1. Cain MK, Zhang Z, Yuan K-H. Univariate and multivariate skewness and kurtosis for measuring nonnormality: Prevalence, influence and estimation. Behavior Research Methods 2016 49:5. 2016;49:1716–35.

2. Kim H-Y. Statistical notes for clinical researchers: assessing normal distribution (2) using skewness and kurtosis. Restorative Dentistry & Endodontics. 2013;38:52.

3. Cook D, Weisberg S. Residuals and Influence in Regression. New York, NY: Chapman and Hall; 1982.
